# Preschool Wheezing and Gastro-Esophageal Reflux: --Causal or Casual Coincidence? Update from Literature

**DOI:** 10.3390/children8030180

**Published:** 2021-02-28

**Authors:** Melissa Borrelli, Giuliana Ponte, Erasmo Miele, Marco Maglione, Carlo Caffarelli, Francesca Santamaria

**Affiliations:** 1 Department of Translational Medical Sciences, Pediatric Section, University of Naples, via Sergio Pansini, 5, 80131 Naples, Italy; melissa.borrelli@unina.it (M.B.); giul.ponte@gmail.com (G.P.); erasmo.miele@unina.it (E.M.); marcomaglione84@gmail.com (M.M.); 2 Department of Medicine and Surgery, University of Parma, via Antonio Gramsci 14, 43126 Parma, Italy; carlo.caffarelli@unipr.it

**Keywords:** gastro-esophageal reflux, preschool, wheezing, children, relationship

## Abstract

Gastroesophageal reflux (GER) and wheeze are two common conditions in children. GER has been advocated as a causative factor for explaining recurrent to persistent respiratory symptoms at any age. This association very often means that many children with cough, wheezing, or recurrent respiratory infections receive empirical anti-reflux medications. The causal relationship is still largely discussed. Compared to the large number of studies in infants and adolescents, literature on the relationship between GER and wheeze in preschool children is scarce and inconclusive. The aim of the present narrative review was to summarize what is known so far, and what the literature has proposed in the last 20 years, on the relationship between preschool wheezing and GER. In preschool children with respiratory symptoms there is a high rate of positivity of reflux testing, for this reason pH-MII testing and endoscopy are recommended. Flexible bronchoscopy may be useful to exclude anatomical abnormalities as the cause of wheezing in infancy and preschool years. Several biomarkers, as well as empirical anti-reflux therapy, have been proposed for the diagnosis of GER-related airway diseases, but the conclusions of these studies are controversial or even conflicting. There is a great need for future clinical trials to confirm or rule out the association.

## 1. Introduction

It is now several years since we started to talk about the relationship between gastro-esophageal reflux (GER) and respiratory disease. At any age, GER may cause a variety of airway symptoms, namely cough, wheeze, and recurrent pneumonia, and also life-threatening events, such as sudden infant death syndrome (SIDS). In particular, in the pediatric population there is great attention to the relationship between reflux and wheezing, as both conditions are very common, may coexist, and have significant effects on the individuals’ health. Many authors emphasize that GER should be sought out as a comorbidity in children with uncontrolled asthma. However, even today the mechanisms underlying extra-esophageal airway manifestations are still poorly understood, and therefore the relationship between the two common conditions could only be a casual coexistence. 

Compared to the large number of studies in infants and adolescents, literature on the relationship between GER and wheeze in preschool children is scarce and inconclusive. 

The aim of the present narrative review was to summarize what is known so far, and what the literature has proposed in the last 20 years, regarding the relationship between preschool wheezing and GER. 

## 2. Methods

**Figure 1:**
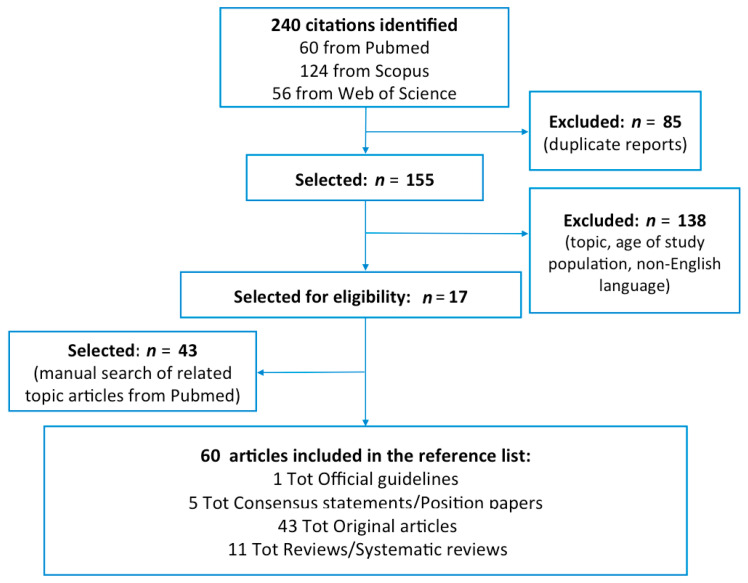
Flow-chart showing selection process of the articles.

We carried out an electronic keyword literature search for English articles published on this topic from January 2000 up to December 2020 in the Scopus, Pubmed, and Web of Science databases. The terms “preschool and wheezing and gastroesophageal reflux” were used as keywords in combination, and the studies obtained were evaluated for selecting relevant literature. This strategy yielded 240 articles, of which 85 duplicates were removed. From the 155 abstracts screened, 138 were excluded because they were not relevant (topic, age, and non-English language). A total of 17 full text articles were assessed for eligibility; together with forty-three additional articles derived from manual Pubmed research focused on our topic. The flow-chart of the study selection process is shown in Figure 1.

### 2.1. Definition of GER 

In 2009, the joint committee of the North American Society for Pediatric Gastroenterology, Hepatology, and Nutrition (NASPGHAN), and the European Society for Pediatric Gastroenterology, Hepatology, and Nutrition (ESPGHAN) published a medical position paper on gastro-esophageal reflux (GER) and GER disease (GERD) in infants and children, based on evidence reviewed from pediatric studies [1]. This document defined GER as the physiologic passage of gastric contents into the esophagus, with or without regurgitation and/or vomiting. GER is considered to be pathologic, and is referred to as GERD when the reflux leads to troublesome symptoms and/or complications. In the most recent clinical practice guidelines [2] the working group adapted this same definition of pediatric GERD for all age groups, despite recognizing a lot of limitations [3]. In the pediatric population, in fact, the symptoms of GERD vary widely and are not specific, and many of them occur in both children with or without GERD, making a definitive diagnosis challenging. Moreover, infant and young children are not able to effectively communicate symptoms, and as a consequence proving that reflux events cause one or multiple symptoms is often difficult. Finally, to date no gold standard diagnostic tool exists for the diagnosis of GERD in infants and children [2]. 

### 2.2. Definition of Preschool Wheeze 

Wheeze is defined as a continuous high-pitched sound, with a musical quality, emitted from the chest during expiration, which probably results from turbulence through narrowed tubes [4]. It is one of a number of forms of noisy breathing in preschool children. Especially if confirmed by a health professional examination, infant and preschool wheeze is associated with lower airway obstruction [5]. Most commonly respiratory symptoms are included in the clinical spectrum of the episodic viral wheeze (EVW), which means that children who start wheezing in conjunction with a viral cold can stop wheezing once the infection passes, and are quite well between episodes [6]. Conversely, other children may require frequent visits because of persistent symptoms during discrete exacerbations and intervals between infections, and this can be due to other triggers such as allergens, cigarette smoke, crying, laughter, or exercise. For this phenotype, the term multiple-trigger wheeze (MTW) was created [6]. Importantly, the likelihood of later developing asthma is much lower in children with EVW compared to those with MTW.

## 3. Results

### 3.1. Prevalence of GER

GER or regurgitation is a common phenomenon in infancy and children. Data from the literature reported a wide variety of percentages, sometimes accounting for serious manifestations of complicated GER, and sometimes limiting the observations to children with one regurgitation episode per day.

Nelson et al. reported that at least one bout of regurgitation per day may be present in 50% of infants aged 0–3 months, with eventually a peak prevalence of approximately 60% between age 4 and 6 months [7]. Beyond that age, a progressive decline is reported, and by 10–12 months, only 5% of infants still have regurgitation. Nevertheless, infants with spilling on 90 days or more during the first 2 years of life are more likely to have GER symptoms at 9 years of age [8]. Using a stricter definition of infant regurgitation, based on the Rome II criteria [9], a lower prevalence (12%) was observed [10].

The prevalence of symptoms of pediatric GERD has also been reported. In a cross-sectional survey of 16 pediatric group practices in the Chicago area, it was reported that symptoms suggestive of GERD are relatively common in children [11]. In particular, parents of 3- to 9-year-old children reported that their kids experienced heartburn, epigastric pain, and regurgitation 1.8%, 7.2%, and 2.3% of the time, respectively, with a slight increase in older children [11]. These findings are consistent with those from a large French study [12]. 

The prevalence of the severe manifestations of GERD in children is unclear. There is a relative scarcity of information on the prevalence of endoscopic findings of GERD among children. The rate of erosive esophagitis increases with age, starting with 5.5% in young children and progressively increasing to 19.6% by age 17. In children aged between 2 and 6 years the prevalence is approximately 7.5% [13].

In conclusion, most infants may have physiological GER that subsides by 18 months of age, but GERD is not as common in early childhood. Conversely, the prevalence of GERD increases with age and, by adolescence, becomes similar to that reported in adults. 

### 3.2. Prevalence of Wheeze

One of the difficulties in defining the true prevalence of wheeze is that the term rarely conveys a specific meaning, not only to lay people, but also to clinicians, within or between cultures [14,15]. 

Many preschool children present to the primary care practitioner with recurrent wheeze, alone or combined with cough [16]. Recurrent wheeze also leads to repeated prescriptions of inhaled drugs (also including steroids and bronchodilators), antibiotics, or cough mixtures, with significant costs to the healthcare system and the family [17].

Nearly 30% of children have at least one episode of wheeze before their third birthday, and by 6 years the prevalence is almost 50% [6]. Frequent wheezing, that is four or more episodes in the previous year [18], is very common among young children, and the prevalence over the first 6 years may rise up to 40% [19]. Regrettably, diagnosing wheezing in preschool children is a challenge that may generate variability in prescription [20].

A cross-sectional survey of a population of children aged 1–5 years in the US and Europe reported that recurrent days with wheeze were present in 42% of cases, and for at least 1 week in one third of these [21]. Actually, the true prevalence of recurrent wheeze is underestimated because the symptom history has the characteristic of being a second-hand description, and the child is often away from the primary caretaker for large parts of the day. However, the identification and follow-up of preschool children with recurrent wheezing is of crucial importance, as the condition is a risk factor for hospitalization due to pneumonia. 

### 3.3. Mechanisms of the Association between GER and Wheeze 

The role of GER as a trigger factor in asthma is an important issue. More than seventy years ago, Mendelson and co-workers called attention to the "acute asthma-like reaction" which follows the aspiration of gastric contents during the induction for anesthesia [22]. The passage of fluids from the stomach into the airways may induce bronchospasm. Macro-aspiration, rare in the absence of an altered level of consciousness, has been shown to cause reflex airway closure associated with chemical damage (such as hemorrhagic pneumonitis and non-cardiac pulmonary edema) [23]. According to the “reflux theory”, micro-aspiration leads to bronchospasm directly through the stimulation of the laryngeal–tracheal receptors [24,25]. As the trachea–bronchial tree and the esophagus recognize common embryonic foregut origins, and share autonomic innervation through the vagus nerve, another potential mechanism is the stimulation of the esophageal mucosal receptors by acidification that activates the vago–vagal reflex and increases bronchial resistance (“reflex theory”) [26]. Several studies, which documented the occurrence of bronchoconstriction after esophageal acidification in patients with asthma, have also shown that atropine inhibited this effect, thus indicating a vagal mediation [27,28]. Moreover, metacholin-induced bronchial hyperreactivity was increased in both adults and children with asthma after intraesophageal administration of acid [29,30].

GER can induce, but also can result from, alteration in the mechanics of breathing. Bronchospasm may, in turn, trigger GER by increasing transdiaphragmatic pressure, thus directly promoting reflux. 

This event is based on the assumption that during airflow obstruction, increased transdiaphragmatic pressure could pump gastric contents into the esophagus. Alternatively, if the diaphragm is directly involved in the maintenance of the anti-reflux barrier, then geometrical flattening of the diaphragm during bronchospasm may adversely affect diaphragmatic performance [26,31]. 

Moreover, predisposing factors for GERD development may include several asthma medications, but their exact role in the occurrence of GER is unclear or even contradictory [32]. The systemic administration of some drugs used to treat bronchospasm, such as beta-2 adrenergic agonists and theophylline, was associated with decreased tone of the lower esophageal sphincter (LES), thus promoting reflux [33]. Conversely, inhalant beta-2 adrenergic agonists, and inhalant and/or oral corticosteroids do not seem to alter the LES tone [29,30]. Nevertheless, in adults with stable, moderate persistent asthma oral corticosteroids increase the esophageal acid contact times at both the distal and proximal pH probes, even in the absence of GER symptoms [34]. 

### 3.4. What Has the Literature Added in the Last Two Decades

Only a few studies have evaluated the prevalence of GER diagnosed by esophageal pH monitoring in preschool children with severe recurrent wheezing, and the values ranged from 70.5% to 85.7% in children aged one to 58 months, and between 18 and 58 months, respectively [35,36]. Similar percentages were observed when the whole pediatric population was evaluated [37,38]. It appears that persistent wheeze has a high specificity for predicting GER, which increased significantly when wheeze was combined with another symptom [36]. A 1.5 times higher risk of wheezing has also been reported in preschool children whose parents used bottle feeding in bed or crib before sleeping time in the first year of life [39]. In these circumstances, frequent episodes of GER and the micro-aspiration of liquids may result in chronic airway inflammation and remodeling that worsen bronchoconstriction, as shown in a murine model [40]. GER is also a likely cause of the severe forms of wheezing which require hospitalization [41].

Even though not necessarily associated with wheezing, another respiratory manifestation possibly related to GER is obstructive sleep apneas (OSA). The functional link between these conditions is still discussed and, even though some reports showed no direct temporal relationship between reflux and apnea episodes that were recorded during polysomnography with pH-metry [42,43], other elements suggest a causal relationship. Among these, the reduction observed in OSA episodes during proton pump inhibitor (PPI) treatment is probably the most relevant [44]. Nevertheless, given the multifactorial etiology of OSAs, particularly in preschool children in whom the leading problem is often represented by oversized tonsils, the role of GER could be of secondary importance. Therefore, efforts to confirm this diagnosis and the need for a treatment should be carefully evaluated in children with OSAs.

Actually, as the association between wheeze and GER does not necessarily indicate an etiological link between the two conditions, several biomarkers have been proposed for diagnosis of GER-related airway diseases. In particular, two bronchoalveolar lavage (BAL) parameters have been previously demonstrated to correlate with severity indices of GER, i.e., the lipid–laden alveolar macrophages (LLAM) and the rate of neutrophilic inflammation, but their specificity and accuracy are questionable [45,46,47]. 

In a retrospective review, sixty children with either chronic or recurrent respiratory symptoms, including chronic wheeze, and a positive esophago–gastric pH-metry who did not respond to respiratory treatment (including bronchodilators, inhaled and systemic steroids, leukotriene modifiers, and antibiotics) or to “anti-reflux” treatment (i.e., lifestyle modifications and pharmacologic treatment) underwent double fiberoptic, airway, and esophago–gastro-duodenoscopies [48]. Inflammatory changes of the airways, such as either positive LLAM or neutrophilic inflammation, were found on BAL samples in over 60% of the cases, also including preschool children [48]. Moreover, it was found that the esophagoscopic findings suggesting GER-related abnormalities, and confirmed histologically by biopsy analysis, were detected only in a low percentage of children. This finding is consistent with the high prevalence of non-acid refluxes observed in children with respiratory symptoms [37,49,50]. 

A high BAL neutrophil count was shown in preschool children with steroid-refractory recurrent wheezing and GER; neutrophilic inflammation of the respiratory airway may be a marker of gastroesophageal reflux [51].

Another proposed marker in the BAL fluid for diagnosing GER-related pulmonary aspiration is pepsin, a proteolytic enzyme secreted by gastric chef cells that usually are not detected in the lower respiratory tract [52]. Currently, BAL pepsin concentrations are significantly higher in wheezy infants with silent reflux and combined multichannel intraluminal impedance testing with pH (pH-MII) positive, and in those with typical GERD symptoms and combined pH-MII negative [53]. At present, no data in preschool children older than 2 years with wheeze are available.

Another biomarker, which, being non-invasive and relatively easy to obtain, is already widely used in pediatric respiratory medicine, is exhaled nitric oxide (eNO). In wheezing infants and toddlers eNO has proven useful in predicting persistence of asthma at school age and lung function decline [54,55], but its correlation with the presence of GER has been poorly investigated. Nevertheless, an altered NO production subsequent to recurrent micro-aspiration has been hypothesized in children with asthma and GER, whose eNO levels were found to be lower compared to asthmatic controls without GER [56]. This finding may be explained by the neutrophilic airway inflammation described in children with GER and wheezing [45], and likely associated with biochemical events not promoting NO production. However, given the limited literature, this biomarker is still far from being diagnostic in children whose wheezing is suspected to be secondary to GER.

Given the high prevalence of GER in wheezing children and the absence of a single diagnostic test, an empirical therapeutic trial as diagnostic confirmation in preschool children with severe, recurrent wheeze has been proposed [35]. As the beneficial effects of anti-reflux therapy on the course of asthma have been emphasized, anti-reflux treatment, mainly with proton pump inhibitors (PPIs), is recommended in all patients with concurrent asthma and GER, irrespective of severity of clinical GER symptoms, and even in those with silent GER [38,57,58,59]. The dosage level and the duration of the therapy have been poorly defined in preschool children, but should be carefully taken into account because of the increased risk of adverse effects, particularly pulmonary infections [60]. Overall, the conclusions from studies on the effects of anti-reflux medical treatment on asthma outcomes in the general pediatric population are contradictory or not convincing. Rosen et al. showed that only a small percentage of children felt there was symptomatic improvement in respiratory symptoms with the administration of a PPI [37]. In accordance with this, a trial in children with poorly controlled asthma with no GERD symptoms showed that PPIs improved neither symptoms nor lung function, but was conversely associated with more respiratory infections [61].

### 3.5. Pulmonologist’s Point of View

The British guidelines on the management of asthma suggest that in wheezy children with clinical clues to alternative diagnoses, including GER, there may be a need to investigate for these conditions, especially when there is insufficient evidence at the initial clinical assessment to make a firm diagnosis of asthma, or an alternative diagnosis is more likely [62]. Accordingly, GER should primarily be excluded when vomiting and/or failure to thrive are reported. A GINA 2020 document recommends considering and excluding alternative causes that can lead to respiratory symptoms such as GER, in particular in children with recurrent chest infections or who present with cough when feeding or vomit after large feeds or have poor response to asthma medications [63]. Moreover, in children and adolescents with asthma, GER and GERD are recognized as comorbidities, that could induce modifiable behaviors. The nature of the association is frankly unclear, and there is no evidence that treatment for GERD improves asthma symptoms in children and adolescents with GERD and asthma [62,63]. 

### 3.6. Gastroenterologist’s Point of View

The NASPGHAN and the ESPGHAN recognize that either upper respiratory tract symptoms and signs such as stridor, cough, and hoarseness, or those deriving from reactive lower airways disease, i.e., asthma and wheezing, may be associated with GER [1,2]. Accordingly, GER is recognized as a contributing or aggravating factor causing wheezing or asthma in selected cases, i.e., those presenting with asthma and associated heartburn or nocturnal symptoms, or frequent asthma exacerbations and continued requirement for short-acting β-agonists, despite inhaled corticosteroids and steroid-dependent difficult-to-control asthma [1]. These symptoms are less reliable in the pediatric population than in adults, therefore experts propose that infants and children undergo esophageal pH monitoring, with or without impedance, before a trial with long-term medical or surgical anti-reflux therapy is prescribed [1]. Furthermore, even though esophageal pH-MII has traditionally represented the technique of choice in children presenting solely with extraesophageal symptoms, there is evidence that, in these patients an esophageal endoscopy with biopsy is also useful [2]. Indeed, up to 32% of children with only cough or other respiratory symptoms may have microscopic esophagitis, and up to 8% may have eosinophilic esophagitis [2].

## 4. Discussion

Wheezing and GER are both common medical conditions and they often co-exist in children. Whether this frequent coexistence derives from a causal relationship is still unclear and, despite a significant body of pediatric literature being published over recent decades, the answer to this question has not been unequivocally provided. Moreover, studies involving preschoolers are even more scarce and inconclusive, thus leaving several areas of uncertainty in the relationship between the two conditions in this age group. 

Studies from the last 20 years have mainly focused: on the assessment of the coexistence of the two conditions by means of the available diagnostic tests [36,37,38,39,45,46,47,52,53,58]; on animal experiments [24,25,40]; or on the response of respiratory symptoms after anti-reflux treatment [61]. Nevertheless, this reasonable approach has not provided convincing evidence of a causal relationship between GER and wheeze. 

There are several reasons for these inconclusive results. First, the definitions of both GER and wheeze are intrinsically elusive and largely rely on symptoms referred by parents. This entails that respiratory noises unrelated to bronchial obstruction may be often misinterpreted as wheeze, and even that clinicians with different experiences may disagree on how to define wheeze. Similarly, discriminating between physiologic GER and a pathological condition is often hard for both parents and clinicians, due to the difficulty in establishing when regurgitation, excessive crying, and other associated symptoms become “troublesome” enough to meet the definition of GERD.

Second, for both conditions, there is a substantial lack of diagnostic tests able to provide reasonable evidence of a cause–effect relationship. PH-MII, widely considered the technique of choice to investigate GERD in children with extra-esophageal manifestations, represents a reliable measure of esophageal exposure to reflux, but even positive results do not allow concluding that wheeze is secondary to GER. Similarly, esophageal endoscopy, which is the gold standard to diagnose lesions secondary to GERD, is not directly informative on the effects of GERD on airways. 

On the other hand, flexible bronchoscopy may be useful for diagnosing the cause of wheezing in children, particularly when anatomical abnormalities such as airway malacia and extrinsic impingement of the airway must be ruled out [62,64,65]. It may also help to characterize pathogens or inflammatory patterns, and to assess whether aspiration is present [66]. Nevertheless, even though the analysis of markers in BAL fluid may be helpful in cases with suspected GERD and inconclusive pH-MII, their variable sensitivity and specificity raises doubts about their reliability as markers of aspiration.

The mentioned limits of our current knowledge on this topic prevent conclusive statements on the causal link between GER and wheeze. Nevertheless, despite the absence of robust evidence, the available literature suggests that a simple coexistence of two unrelated conditions is unlikely. Of course, this does not entail a cause–effect relationship in all wheezing children, but, moving from daily clinical practice, the possibility that GER may play a role in exacerbating the respiratory symptoms of selected patients, at least as a cofactor, appears concrete. The impact of GER on wheeze is likely to vary deeply from case to case, thus making it difficult to generalize recommendations on when and how to investigate and treat GER in a wheezing preschooler. 

These speculations should never prevent clinicians from performing a thorough evaluation of all wheezing preschoolers, taking into account differential diagnoses and other exacerbating factors. Nevertheless, when patients show poor response to standard therapy or when a suggestive clinical picture is present, the possibility that GER may be playing a role should be kept in mind, in order to consider potentially beneficial therapeutic options.

In conclusion, many questions remain unanswered about the etiologic association between GER and wheezing in preschool children, and the conclusions from the available studies are controversial, or even conflicting. Therefore, given the likelihood of something more than a simple casual coexistence, solid clinical trials to confirm or rule out an etiologic association are urgently needed.

## Data Availability

No new data were created or analyzed in this study. Data sharing is not applicable to this article.
